# Draft Genome Sequence of *Actinomyces* *succiniciruminis* Strain Am4^T^, Isolated from Cow Rumen Fluid

**DOI:** 10.1128/genomeA.01587-16

**Published:** 2017-07-20

**Authors:** Susakul Palakawong Na Ayudthaya, Bastian Hornung, Adithi Ravikumar Varadarajan, Wendy Plugge, Caroline M. Plugge

**Affiliations:** a Laboratory of Microbiology, Wageningen University, Wageningen, The Netherlands; b Laboratory of Systems and Synthetic Biology, Wageningen University, Wageningen, The Netherlands

## Abstract

*Actinomyces succiniciruminis* strain Am4^T^, isolated from cow rumen fluid, can metabolize a range of substrates including complex carbohydrates to organic acids. Here, we report a 3.33-Mbp draft genome of *Actinomyces succiniciruminis*.

## GENOME ANNOUNCEMENT

The genus *Actinomyces* is comprised of many species that can be found in various environments. Here, we report the draft genome sequence of a species belonging to the genus *Actinomyces* that originates from a cow rumen. *Actinomyces succiniciruminis* (Am4^T^) was isolated from an enrichment of amylopectin using rumen microorganisms (1).

The genus *Actinomyces* is one of the largest genera within the order and class *Actinobacteria* (2). This genus currently contains 47 species and two subspecies that are Gram-positive, pleomorphic, nonmotile, anaerobic, and aerotolerant bacteria with high G+C content (2). *Actinomyces succiniciruminis* can convert amylopectin, starch, and starch waste to mainly succinate, lactate, and small amounts of acetate and formate (1).

Genomic DNA of strain Am4^T^ was extracted from glucose grown cells (1) using the MasterPure complete DNA and RNA purification kit (Epicenter, Madison, WI). Genome sequencing was performed on an Illumina MiSeq sequencer with a read length of 250 bp and an insert size of 500 at GATC-Biotech, Konstanz, Germany. The genome size was first estimated by using kmerspectrumanalyzer (3) on the complete left side of the paired-end data. The quality of the reads was evaluated by FastQC (http://www.bioinformatics.babraham.ac.uk/projects/fastqc/). The reads were trimmed and then the adapters were removed using Trimmomatic, version 0.32 (4). Afterward the assembly was performed using Velvet v1.2.10 (5), with a k-mer value of 41 and a minimum contig size of 500, followed by gapfilling with Gapfiller v1.11 (6). Annotation was carried out with an in-house pipeline, as described in references 7, 8, followed by additional annotation via PRIAM (9), version March 2013. Mapping of reads was performed with BWA v0.7.7 (10). Identification of CAZymes was performed via dbcan v3.0 (11).

The total draft genome consists of 3.33 Mbp and 91 scaffolds with an *N*_50_ of 69,311 bp and a G+C content of 69.8%, which includes 99.37% of all filtered reads. The genome contains 50 tRNAs genes, one of each rRNA subunit and encodes 2,897 putative proteins, of which 832 were denoted as hypothetical.

Of the 2,897 proteins, 1,745 could be classified according to the COG database (12). Of these 1,745 proteins, 273 are involved in carbohydrate transport and metabolism, followed by 245 with only a predicted function and 180 for amino acid transport and metabolism.

Metabolic mapping with Pathway Tools v17 (13) showed that the organism is capable of degrading glucose, galactose, mannose, and maltose. Furthermore, 173 CAZymes were identified. They enable the organism to degrade complex carbohydrates like starch or glycogen. Multiple enzyme coding genes were presented with multiple copies, e.g., eight beta-glucosidases, 21 glycosyltransferases type 4, four alpha-l-rhamnosidases, 16 glycosylhydrolases type 13, including alpha-amylases, oligo-1,6-glucosidases, pullulanases, and neopullulanase, showing the broad capacity of *A. succiniciruminis* (Am4^T^) to degrade complex carbohydrates.

### Accession number(s).

The draft genome sequence of *Actinomyces succiniciruminis* strain Am4^T^ has been deposited at DDBJ/EMBL/GenBank under the accession no. LK995455 to LK995545. The version described in this paper is the first version.
